# 1-year follow-up of neurofeedback treatment in adolescents with attention-deficit hyperactivity disorder: randomised controlled trial

**DOI:** 10.1192/bjpo.bp.115.000166

**Published:** 2016-03-02

**Authors:** Marleen Bink, Ilja L. Bongers, Arne Popma, Tieme W.P. Janssen, Chijs van Nieuwenhuizen

**Affiliations:** **Marleen Bink**, PhD, Department of Clinical Neuropsychology, VU University Amsterdam, Amsterdam; Scientific Center for Care & Welfare (Tranzo), Tilburg University, Tilburg, The Netherlands; **Ilja L. Bongers**, PhD, GGzECenter for Child and Adolescent Psychiatry, Eindhoven; Scientific Center for Care & Welfare (Tranzo), Tilburg University, Tilburg, The Netherlands; **Arne Popma**, MD, PhD, Academic Department of Child & Adolescent Psychiatry, VUmc/De Bascule, Duivendrecht, The Netherlands; **Tieme W. P. Janssen**, MSc, Department of Clinical Neuropsychology, VU University Amsterdam, Amsterdam, The Netherlands; **Chijs van Nieuwenhuizen**, PhD, Scientific Center for Care & Welfare (Tranzo), Tilburg University, Tilburg; GGzECenter for Child and Adolescent Psychiatry, Eindhoven, The Netherlands

## Abstract

**Background:**

Estimates of the effectiveness of neurofeedback as a treatment for attention-deficit hyperactivity disorder (ADHD) are mixed.

**Aims:**

To investigate the long-term additional effects of neurofeedback (NFB) compared with treatment as usual (TAU) for adolescents with ADHD.

**Method:**

Using a multicentre parallel-randomised controlled trial design, 60 adolescents with a DSM-IV-TR diagnosis of ADHD receiving NFB+TAU (*n*=41) or TAU (*n*=19) were followed up. Neurofeedback treatment consisted of approximately 37 sessions of theta/sensorimotor rhythm (SMR)-training on the vertex (Cz). Outcome measures included behavioural self-reports and neurocognitive measures. Allocation to the conditions was unmasked.

**Results:**

At 1-year follow-up, inattention as reported by adolescents was decreased (range *η*_p_^2^=0.23–0.36, P<0.01) and performance on neurocognitive tasks was faster (range *η*_p_^2^=0.20–0.67, *P*<0.005) irrespective of treatment group.

**Conclusions:**

Overall, NFB+TAU was as effective as TAU. Given the absence of robust additional effects of neurofeedback in the current study, results do not support the use of theta/SMR neurofeedback as a treatment for adolescents with ADHD and comorbid disorders in clinical practice.

**Declaration of interest:**

None.

**Copyright and usage:**

© The Royal College of Psychiatrists 2016. This is an open access article distributed under the terms of the Creative Commons Non-Commercial, No Derivatives (CC BY-NC-ND) licence.

Attention-deficit hyperactivity disorder (ADHD) is characterised by the re-occurring patterns of inattention and/or hyperactivity/impulsivity symptoms that interfere with developmentally appropriate social, academic or occupational functioning.^1^ Neurodevelopmental conditions such as intellectual disabilities, conduct disorder, depression and anxiety are seen more often in youngsters with ADHD than in youngsters without ADHD.^2^ In addition, it is estimated that around a third of youngsters with autism spectrum disorder (ASD) display ADHD comorbidity.^3^ Stimulant medication and behavioural therapy are considered the treatments of choice for ADHD. Stimulant medication is effective in reducing ADHD symptoms in youngsters with ADHD^4^ and – although possibly to a lesser extent – in youngsters with combined ADHD and ASD.^5^ Although medication seems to be effective, there are limitations as well, such as limited knowledge of long-term efficacy and side-effects.^6^ Moreover, despite the persistent nature of ADHD, the majority of adolescents with ADHD discontinue stimulant medication before adulthood.^7^ Additions or alternatives to the current treatment as usual (TAU) to reduce ADHD symptoms further, and on a long-term basis, are therefore desirable. Neurofeedback has been suggested as a potentially effective intervention for reducing ADHD^8^ and ASD^9^ symptoms.

Neurofeedback is intended to alter brain activity by providing feedback from electroencephalogram (EEG) activity to patients; this is expected to lead to improvements in behaviour. Overall, youngsters with ADHD show increased theta^10,11^ and decreased beta^11^ activity compared with typically developing youngsters. Increased theta (4–7 Hz) is associated with lower vigilance, and decreased beta (13–30 Hz) is associated with reduced attention.^12^ In addition, the sensorimotor rhythm (SMR; 12–16 Hz), measured above the central sulcus, has been related to behavioural inhibition.^13^ As neurofeedback aims to reduce ADHD symptoms such as diminished vigilance, attention and inhibition, most neurofeedback protocols train suppression of theta activity and reinforcement of beta (12–20 Hz) or SMR (12–15 Hz) with electrode placement on the vertex (Cz).^8,10^ A complete neurofeedback intervention typically comprises 20–40 training sessions.^10^ The effectiveness of neurofeedback as treatment for ADHD is still actively debated.

Estimates of the effectiveness of neurofeedback for the treatment of ADHD range from efficacious^14^ to non-significant when only probably masked studies are considered.^15^ It has been suggested that a major advantage of neurofeedback over medication may be long-term effects after treatment completion. To date there has been only one randomised controlled trial (RCT) comparing the long-term effects of neurofeedback and stimulant medication.^16^ This study demonstrated similar levels of ADHD symptoms at 6-month follow-up for children who had received neurofeedback (*n*=12) and children treated with stimulant medication (*n*=11). It should be noted that at the 6-month follow-up, 8 of the 12 children who had received neurofeedback had started stimulant medication treatment.^16^ Long-term effects of neurofeedback were found in two RCT studies: neurofeedback was more effective in reducing ADHD symptoms as reported by parents than computerised attention training up to 6 months of post-treatment as stand-alone treatment^17^ or add-on treatment.^18^

In summary, neurofeedback is viewed as a potentially effective treatment for ADHD symptoms. Comparative studies of short-term effects have shown that neurofeedback training can be as effective as stimulant medication,^16,19^ but evidence on the longterm effects of neurofeedback is limited. Since the aim of neurofeedback is to induce enduring changes in brain regulation to improve behaviour, we predict long-term effects as a consequence of improved brain functioning. The aim of the current study was therefore to investigate the value of neurofeedback as a supplement to TAU for adolescents with ADHD and comorbid disorders at 1-year follow-up.

## Method

### Participants

Eligible participants were male adolescents with Dutch as their native language, aged between 12 and 24 years, with a primary clinical DSM-IV-TR^1^ diagnosis of ADHD and a full-scale total intelligence quotient (TIQ) >80 on the Wechsler Intelligence Scale for Children (WISC-III) or the Wechsler Adult Intelligence Scale (WAIS-III).^20,21^ Adolescents diagnosed with ASD (autism, Asperger syndrome or pervasive developmental disorder not otherwise specified (PDD-NOS)) with confirmed clinical ADHD symptoms sufficient for a clinical diagnosis were also included. ADHD symptoms were verified by a Dutch semi-structured DSM-IV-based ADHD interview for adults^22^ and the Mini International Neuropsychiatric Interview (MINI).^23,24^ Exclusion criteria were neurological disorders, schizophrenia and other psychotic disorders.

**Fig. 1:**
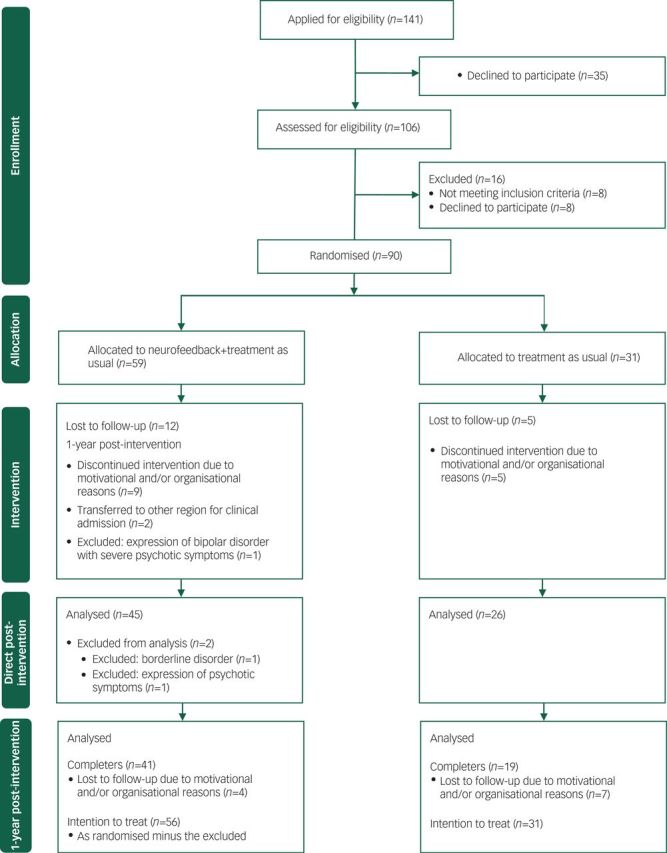
Participant flow diagram.

In total 90 adolescents were randomly assigned to the treatment groups: combined neurofeedback (NFB) and TAU (NFB+TAU group; *n*=59) or TAU only (TAU group; *n*=31). The total drop-out and exclusion rate after randomisation did not differ for the NFB+TAU group (*n*=18, 30.5%) or the TAU group (*n*=12, 38.7%), *P*=0.485 two-tailed Fisher’s exact test. The participant flow diagram is presented in Fig. 1.

Medication use and comorbid disorders were allowed. Comorbid disorders present in the final group at 1-year follow-up were: depressive disorders (*n*=1), anxiety disorders (*n*=3), substance-related disorders (*n*=3), conduct disorders (*n*=3), learning disorders (*n*=5), tic disorders (*n*=1) and reactive attachment disorder (*n*=1). The final group characteristics are listed in Table 1.

**Table 1:** Group characteristics

	Total	NFB+TAU	TAU
	*n*=60	*n*=41	*n*=19
Age at *T*_1_, years: mean (s.d.)	15.95 (3.33)	15.85 (3.34)	16.16 (3.40)

DSM-IV-TR, *n* (%)			
Diagnosis ADHD	38 (63)	26 (63)	12 (63)
Diagnosis ASD+ADHD	22 (37)	15 (37)	7 (37)

Treatment as usual, *n* (%)			
Stimulant medication *T*_1_	31 (52)	19 (46)	12 (63)
Stimulant medication *T*_2_	29 (48)	17 (41)	12 (63)
Stimulant medication *T*_3_	30 (49)	19 (46)	11 (58)
Behavioural interventions,^a^ *n* (%)			
Adolescent *T*_1_–*T*_2_	22 (37)	13 (32)	9 (47)
Adolescent *T*_2_–*T*_3_	13 (22)	9 (22)	5 (26)
Adolescent *T*_1_–*T*_3_	26 (43)	16 (39)	10 (53)
Parent *T*_1_–*T*_2_	16 (27)	11 (27)	5 (26)
Parent *T*_2_–*T*_3_	6 (10)	5 (12)	1 (5)
Parent *T*_1_–*T*_3_	18 (30)	12 (29)	6 (32)

Childhood behaviour, mean (s.d.)			
ADHD rating: inattention^b^	6.10 (2.46)	5.85 (2.74)	6.63 (1.67)
ADHD rating: hyperactivity/impulsivity^b^	4.83 (2.80)	4.71 (2.87)	5.11 (2.69)

Intelligence, total IQ: mean (s.d.)	101.12 (11.51)	99.22 (10.61)	105.21 (12.57)

*T*_1_, pre-intervention; *T*_2_, direct post-intervention; *T*_3_, 1-year follow-up; NFB, neurofeedback; TAU, treatment as usual; ADHD, attention-deficit hyperactivity disorder; ASD, autism spectrum disorder; GAF, Global Assessment of Functioning.

a Behavioural interventions followed between pre- and post-intervention (*T*_1_–*T*_2_), between post-intervention and the 1-year follow-up (*T*_2_–*T*_3_) and between pre-intervention and 1-year follow-up (*T*_1_–*T*_3_).

b ADHD-rating scale retrospective self-reported childhood symptoms for inattention and hyperactivity/impulsivity. Group characteristics did not differ between groups.

### Trial design

A multicentre parallel group study was conducted, with stratification for age group (12–15 years, 16–20 years and 21–24 years) and unbalanced randomisation to treatment (2 NFB+TAU: 1 TAU). Unbalanced treatment was applied to encourage study participation by greater odds to receive neurofeedback at study entree. The randomisation process was computer-controlled,^25^ using randomly varying block lengths of 3, 6, 9 and 12. An independent administrative employee was responsible for the assignment of participants to the treatment groups immediately after pre-intervention assessment. The participant (and if applicable, his parents) was notified the same day whether he would receive neurofeedback treatment. Allocation to the conditions was unmasked; participants, parents, neurofeedback trainers, the outcome assessor and clinical professionals were aware of group allocations. Different individuals fulfilled the roles of outcome assessor and neurofeedback trainer. All data entry was masked to group allocation (NFB+TAU or TAU) and was checked twice by different research assistants.

Before the study, calculation with G*power version 3.1.5.1^26^ predicted that a total sample size of 46 would be sufficient to detect a medium effect size (*f*=0.25) in a repeated measures ANOVA with two measurements, with an alpha of 0.05 and a power of 90%. In addition, a total sample size of 42 would be sufficient to detect a medium effect size (*f*=− 0.25) in a repeated measures ANOVA with three measurements (pre-intervention, post-intervention and 1-year follow-up), with an alpha of 0.01 and a power of 90%. We have followed the CONSORT 2010 guidelines^27^ for reporting parallel group randomised trials. This trial is registered in the Dutch trial register (Ref. No. 1759, www.trialregister.nl/trialreg/admin/rctview.asp?TC=1759).

### Interventions

#### TAU

Participants received treatment as prescribed by the main therapist in the three participating centres for child and adolescent psychiatry (GGzE, GGzBreburg, Reinier van Arkel group). TAU was monitored using an intervention questionnaire based on the Dutch national basic programme ADHD for children and adolescents.^28^ Behavioural interventions included regular cognitive–behavioural therapy, systemic therapy and/or supportive counselling for the adolescent and/or his parent(s) (Table 1). Stimulants prescribed included immediate release methylphenidate, sustained release methylphenidate and dexamphetamine. One participant used dexamphetamine at study entry and direct post-intervention but changed to sustained release methylphenidate at 1-year follow-up. Two participants were taking atomoxetine at study entry; one participant had changed to sustained release methylphenidate by the time of the 1-year follow-up, and the other participant continued to use atomoxetine through the 1-year follow-up. Because the clinical effects of stimulant medication and atomoxetine are suggested to be similar, this participant was categorised as stimulant-medicated for the purposes of analysis at 1-year follow-up. There were no group differences (TAU *v*. NFB+TAU) in the use of stimulant medication or the type of behavioural therapy received (Table 1).

#### NFB+TAU

Neurofeedback training was carried out over a period of around 5 months (25 weeks), with two to three training sessions every week. Each participant was offered a total of 40 30-minute training sessions. The mean number of training sessions received was 38 (s.d.=4.43), with a minimum of 19 sessions for the adolescents in the NFB+TAU group at 1-year follow-up (*n*=41). A theta/SMR training^29^ – a form of theta/beta training – was used, with thresholds to inhibit theta/alpha frequency bands (4–7 and 8–11 Hz), reward SMR activity (13–15 Hz) and inhibit beta/gamma (22–36 Hz) conducted on Cz. The intervention conditions are described in more detail elsewhere.^30^

### Outcome measures

Outcome measures included three behavioural self-reports as primary outcome measures and four neurocognitive secondary outcome measures.

#### Primary behavioural outcome measures

Primary outcome measures included the following.

- The MINI:^23,24^ ADHD subscales for children and adolescents covering inattention and hyperactivity/impulsivity symptoms over the last 6 months (scale range: 0–9).
- The ADHD rating scale, a DSM-IV-based self-report for adults^31,32^ (adapted from DuPaul *et al*^33^), with subscales for inattention and hyperactivity/impulsivity (scale range: 0–9).^34,35^
- The Youth Self Report (YSR):^36^ the attention problems subscale, the externalising problems scale and total problems scale were used. Participants aged over 18 years also completed the YSR, as most of them were still attending school and living with their parents.

#### Secondary neurocognitive outcome measures

Secondary outcome measures included the following.

- The D2 Attention and Concentration Test^37^ raw scores for total processed items and total correctly processed items.
- The digit span backwards (DSB):^30^ total score (number of rows recalled correctly) and the maximum correctly recalled row length.
- The Stroop Color-Word Test^38,39^ for total execution time of the colour-word card and interference time.
- The Tower of London (TOL) test:^40^ for the raw total correct, total moves, initiation time, execution time and total time were calculated.

### Procedure

Before the start of the study, approval was obtained from the Medical Ethics Committee for Mental Health Institutions in The Netherlands (Ref. no: NL 24776.097.08 CCMO). The study took place in three centres for child and adolescent psychiatry (GGzE, GGzBreburg, Reinier van Arkel group) in the south of The Netherlands. After the study was explained (verbally and in writing), written informed consent was obtained from each participant. For those younger than 18 years, parents also provided written informed consent.

At pre-intervention, participants were seen on three occasions for the administration of behavioural questionnaires, neurocognitive tests, the WAIS or WISC intelligence test and EEG measurements, where applicable medication was taken as normal on the day of assessment. Interventions took place between December 2009 and July 2012. The duration of the intervention period was approximately 25 weeks. Data collection continued until August 2013. Post-intervention and 1-year follow-up assessments included behavioural questionnaires and neurocognitive tests for all 60 participants. There were missing data for the D2 Attention and Concentration Test (two participants), Stroop test (two participants), TOL (one participant) either because of administrative problems or because the participant refused to complete the task. Parent reports (Child Behavior Checklist^41^ and Autism Spectrum Quotient^42^) were not included because of the low response rate (48%) of the total randomised sample at the follow-up measurements.

### Statistical analysis

All analyses were performed by using SPSS version 19.0. Differences in group characteristics were analysed with a one-way ANOVA or a chi-squared test (*χ*^2^) with Fisher’s exact correction. Attrition analyses for behavioural data with smaller sample size than the total sample size due to missing or incomplete data were performed by comparing group characteristics and other pre-intervention primary behavioural measures for the analysed subsample and the total sample using a one-way ANOVA. Differences were considered significant at *P*<0.05 for the group characteristics and the attrition analyses.

Complete case analysis was performed for participants who finished all assessments up to 1 year after the intervention (including neurofeedback training, if applicable), to determine whether neurofeedback had additional value after completion of the training. The effect of neurofeedback training was investigated using a generalised linear model (GLM) with between- and within-participant factors. To control for multiple tests, effects were considered significant at *P*<0.01. This analysis was performed separately for all outcome measures with treatment group as between-participants factor and time (i.e. pre-intervention (*T*_1_), post-intervention (*T*_2_) and 1-year follow-up (*T*_3_)) as within-particpant factor. The full factorial models were tested. All behavioural effects were evaluated using multivariate test criteria. The adjusted difference and 99% confidence interval (99% CI), for the total sample (NFB+TAU and TAU) are reported.

To determine whether changes over time were associated with either the co-occurrence of ASD or stimulant medication use, *post hoc* analyses were performed to look for three-way interactions involving diagnostic group (ADHD or ASD with comorbid ADHD) or stimulant medication use (stimulant-medicated and stimulant-free) at 1-year follow-up as an additional between-participant factor.

Intention-to-treat (ITT) analyses were performed to control for potential outcome bias of individuals who withdrew (*n*=27) after randomisation. Separate analyses were performed with ITT analyses based on imputation with last observation carried forward (LOCF) and ITT analyses based on imputation with expectation maximisation (EM). All analyses were performed for the total group as randomised with the exception of the three excluded participants (see also flowchart, Fig. 1).

## Results

### Group characteristics

There were no baseline differences for the participants who completed the study up to 1 year post-treatment in group characteristics between the NFB+TAU group and the TAU group (Table 1). There were also no pre-intervention group differences on behavioural or neurocognitive measures.

There were no significant differences between the treatment groups in terms of stimulant medication use at pre-intervention or 1-year follow-up. One participant used atomoxetine and stayed on a stable dose of 60 mg throughout the study. For those who used immediate or sustained release methylphenidate at 1-year follow-up (*n*=29), a slight increase in dose was observed from pre-intervention (mean 30.83, s.d.=23.39) to 1-year follow-up (mean 37.81, s.d.=18.69; AD_T3-T1_=8.33, 95% CI 0.07–16.59, *F*(1,27)=4.28, *P*=0.048, *η*_p_^2^=0.14). There were no significant interactions between time and treatment group for dosage, *F*(1,27)=1.92, *P*=0.177, *η*_p_^2^=0.07. In addition, those who used medication at 1-year follow-up (*n*=30) started stimulant medication at 33 months (s.d.=32.39, 95% CI 20.87–45.06) before 1-year follow-up with no differences between treatment groups, *F*(1,28)=0.73, *P*=0.400.

### Attrition analysis

Attrition analysis showed that the participants who dropped out for motivational or organisational reasons (*n=*27) did not differ from the completers group (*n*=60) in terms of group characteristics or behavioural measures at pre-intervention. In addition, the subsamples for the D2 Attention and Concentration Test (*n*=58) and the Stroop test (*n*=57) did not differ from the completers sample (*n*=60) in terms of group characteristics, or behavioural and neurocognitive measures at pre-intervention.

### Complete case analyses

Behavioural and neurocognitive outcome measures are summarised in Table 2.

**Table 2:** Complete case analyses for behavioural and cognitive measures

	Pre-intervention (*T*_1_)		Post-intervention (*T*_2_)		1-year follow-up (*T*_3_)		Adjusted difference	Adjusted difference [99% CI] at	ANOVA Time (*T*_1_–*T*_3_)^b^			ANOVA NFB+TAU & TAU Over time (*T*_1_–*T*_3_)^c^		
					
	NFB+TAU	TAU	NFB+TAU	TAU	NFB+TAU	TAU	(99% CI) at 1-year	1-year follow-up						
	Mean (s.d.)	Mean (s.d.)	Mean (s.d.)	Mean (s.d.)	Mean (s.d.)	Mean (s.d.)	follow-up (*T*_3_–*T*_1_)^a^	(*T*_3_–*T*_2_)^a^	*F*	*η*_p_^2^	*P*	*F*	*η*_p_^2^	*P*
*Behaviour* ^d^														
MINI^e^									*df (1,57)*					
Inattention	5.46 (2.42)	6.32 (2.60)	n/a	n/a	4.12 (2.56)	4.89 (2.74)	−*1.38 (*−*2.26 to* −*0.50)*	n/a	17.53	0.23	*0.000*	0.01	0.00	0.904
Hyperactivity/impulsivity	4.17 (2.64)	3.26 (2.13)	n/a	n/a	2.90 (2.30)	2.74 (2.33)	−*0.90 (*−*1.73 to* −*0.06)*	n/a	8.23	0.12	*0.006*	1.41	0.02	0.241
ADHD-rating^f^									*df (2,57)*					
Inattention	4.63 (2.41)	5.42 (2.04)	2.95 (2.63)	4.00 (2.31)	2.73 (2.32)	4.05 (2.84)	−*1.64 (*−*2.44 to* −*0.83)*	−0.08 (−0.72 to 0.61)	15.80	0.36	*0.000*	0.40	0.01	0.670
Hyperactivity/impulsivity	3.56 (2.12)	2.95 (1.87)	2.49 (2.20)	2.53 (2.39)	2.05 (2.22)	2.53 (2.27)	−*0.97 (*−*1.77 to* −*0.16)*	−0.22 (−0.89 to 0.45)	5.12	0.15	*0.009*	1.62	0.05	0.207
YSR^g^									*df (2,57)*					
Attention	9.63 (3.18)	10.00 (3.18)	7.61 (3.51)	9.63 (3.73)	7.44 (3.46)	7.74 (3.62)	−*2.23 (*−*3.37 to* −*1.08)*	−*1.03 (*−*1.96 to* −*0.11)*	14.02	0.33	*0.000*	3.38	0.11	0.041
Externalising	16.00 (9.99)	14.37 (8.19)	13.80 (7.39)	12.32 (8.08)	12.95 (8.11)	11.11 (7.10)	−*3.16 (*−*5.91 to* −*0.40)*	−1.03 (−3.89 to 1.03)	4.67	0.14	0.013	0.02	0.00	0.974
Total	48.80 (22.50)	53.16 (20.61)	41.10 (18.17)	45.84 (20.17)	39.76 (20.72)	37.68 (18.56)	−*12.26 (*−*19.45 to* −*5.07)*	−4.75 (−10.59 to 1.09)	10.19	0.26	*0.000*	1.28	0.04	0.287
*Cognition*														
D2^h^									*df (2,55)*					
Total processed items	397.79 (59.97)	419.68 (64.89)	451.00 (72.39)	447.26 (82.86)	473.33 (67.40)	472.58 (82.17)	*64.22 (45.09 to 83.34)*	*23.83 (6.39 to 41.26)*	42.48	0.61	*0.000*	2.42	0.08	0.098
Total correct processed items	155.15 (22.77)	162.79 (25.69)	178.87 (29.21)	178.89 (33.04)	185.49 (27.39)	191.89 (39.63)	*29.72 (21.73 to 37.71)*	*9.81 (1.99 to 17.62)*	56.25	0.67	*0.000*	1.28	0.04	0.286
DSB^i^									*df (2,57)*					
Total score	6.51 (1.63)	6.79 (2.15)	6.85 (1.56)	7.53 (2.22)	6.88 (2.08)	7.63 (2.67)	0.60 (−0.12 to 1.33)	0.07 (−0.57 to 0.70)	3.10	0.10	0.053	0.45	0.02	0.640
Longest row	4.76 (0.89)	5.05 (1.08)	4.88 (0.90)	5.16 (1.21)	4.88 (1.19)	5.16 (1.21)	0.25 (−0.17 to 0.66)	13 (−0.26 to 0.53)	1.23	0.04	0.301	0.47	0.02	0.630
Stroop^j^									*df (2,54)*					
Colour-word	100.41 (22.98)	102.32 (28.91)	92.85 (21.10)	99.21 (36.62)	95.15 (29.68)	92.68 (19.55)	−8.16 (−17.09 to 0.78)	−2.98 (−11.56 to 5.59)	3.16	0.10	0.050	1.35	0.05	0.268
Interference	34.46 (14.63)	36.84 (20.22)	30.74 (13.29)	32.26 (22.86)	33.28 (21.48)	30.68 (13.23)	−4.06 (−12.30 to 4.18)	0.10 (−7.87 to 8.07)	1.70	0.06	0.192	0.45	0.02	0.637
														
TOL^k^									*df (2,56)*					
Correct score	3.51 (1.83)	3.67 (1.72)	3.44 (1.85)	3.83 (2.07)	3.59 (2.23)	4.00 (2.50)	0.20 (−0.73 to 1.14)	0.16 (−0.60 to 0.91)	0.19	0.01	0.825	0.10	0.00	0.906
Move score	31.90 (16.48)	31.89 (15.51)	29.83 (15.19)	32.06 (15.95)	31.02 (18.91)	24.50 (12.31)	−4.13 (−11.21 to 3.04)	3.18 (−9.61 to 3.25)	1.24	0.04	0.296	1.62	0.05	0.208
Initiation time	20.49 (10.67)	19.50 (10.37)	20.39 (19.04)	21.83 (13.73)	24.68 (15.25)	26.78 (24.35)	5.74 (−0.12 to 11.60)	4.62 (−1.59 to 10.83)	3.42	0.11	0.040	0.32	0.01	0.727
Execution time	175.78 (70.92)	173.11 (49.61)	142.49 (40.20)	158.78 (47.79)	143.10 (55.32)	132.56 (33.17)	−*36.62 (*−*62.56 to* −*10.68)*	−12.81(−35.85 to 10.24)	7.10	0.20	*0.002*	1.29	0.04	0.283
Total time	196.27 (75.97)	192.61 (50.43)	162.88 (44.11)	180.61 (48.23)	167.78 (60.55)	159.33 (39.71)	−30.88 (−58.69 to −3.07)	−8.19 (−33.21 to 16.83)	4.64	0.14	0.014	1.13	0.04	0.329

NFB, neurofeedback; TAU, treatment as usual; MINI, Mini International Neuropsychiatric Interview; ADHD, attention-deficit hyperactivity disorder; YSR, Youth Self Report; D2, D2 Attention and Concentration Test; DSB, digit span backwards; TOL, Tower of London test.

a Adjusted difference between 1-year follow-up minus pre-intervention are displayed for the total group (NFB+TAU and TAU).

b Generalised linear model ANOVA with time (pre-intervention (*T*_1_) to direct post-intervention (*T*_2_) to 1-year follow-up (*T*_3_)) as within factor.

c Generalised linear model ANOVA with time (*T*_1_–*T*_2_–*T*_3_) as within factor and treatment group (NFB+TAU or TAU) as between factor.

d NFB+TAU (*n*=41) and TAU (*n*=19).

e MINI inattention and hyperactivity/impulsivity were only reported at pre-intervention (T1) and at 1-year follow-up (T3).

f ADHD rating scale self-reported current symptoms for inattention and hyperactivity/impulsivity.

g YSR scales: attention problems, externalising problems and total problems.

h NFB+TAU (*n*=39) and TAU (*n*=19).

i NFB+TAU (*n*=41) and TAU (*n*=19).

j Stroop is measured in seconds, NFB+TAU (*n*=39) and TAU (*n*=18).

k NFB+TAU (*n*=41) and TAU (*n*=18).

Italic values denote *P*<0.01.

#### Primary behavioural outcome measures

All behavioural measures showed reductions in reported inattention and hyperactivity/impulsivity over time, irrespective of treatment group. There were no interactions between treatment group and time.

#### Secondary neurocognitive outcome measures

There were no significant interactions between treatment group and time on the neurocognitive measures. Adolescents showed on the D2 Attention and Concentration Test an increase in total processed items and total correctly processed items from pre-intervention to 1-year follow-up. Similarly, at the 1-year follow-up adolescents were faster on the TOL with shorter execution times than at pre-intervention.

### Comorbidity of ASD and stimulant medication use

#### Comorbidity of ASD

*Post hoc* analysis showed no differences over time between adolescents with ADHD or combined ASD+ADHD. Furthermore, there were no interactions between comorbidity of ASD, time and treatment group.

#### Stimulant medication use

Stimulant-medicated and stimulant-free adolescents did not differ over time on the behavioural and cognitive measures. Neither were there interactions between stimulant medication use, time and treatment group.

### ITT analyses

ITT analyses to control for potential outcome bias due to drop-out based on LOCF as well as EM showed behavioural and neurocognitive outcomes comparable to the complete case analyses. There was a decrease of behavioural problems and faster performance on neurocognitive tasks over time for all adolescents (*n*=87), irrespective of treatment group (NFB+TAU or TAU). The trend (0.01<*P*<0.05) found in the complete case analysis for the YSR attention problems subscale (Table 2) did not persist in the ITT analyses based on LOCF or EM. See supplementary Tables DS1 and DS2.

## Discussion

This study investigated the long-term behavioural and neurocognitive effects of supplementing TAU with neurofeedback, using a multicentre parallel RCT design. Long-term additional effects of neurofeedback up to 1 year post-intervention have not been studied before. Overall, the adolescents reported reductions in ADHD symptoms, irrespective of whether they had received neurofeedback. Similarly, neurocognitive measures of attention and processing speed showed improvements between pre-intervention and follow-up indexed by decreased execution time on the TOL task and increased total processed items on the D2 Attention and Concentration Test, irrespective of received treatment.

There was no additional benefit from supplementing TAU with neurofeedback 1 year after treatment. Previously published results from this RCT showed that adolescents were able to learn to decrease theta activity within the last five training sessions compared with the first five training sessions of neurofeedback.^43^ However, these learning effects did not result in direct additional value of neurofeedback over TAU on behaviour^43^ or cognition.^30^ Our findings are consistent with another RCT^16^ that found similar improvements in stimulant-medicated children and children who had also received neurofeedback. Although Meisel *et al*^16^ did not directly explore the additional value of neurofeedback over TAU, the naturalistic follow-up is reasonably comparable to the current study given the fact that 8 of the 12 children who had received neurofeedback had started stimulant medication treatment and thus can be considered as a combination group of TAU and neurofeedback.

In contrast to the current study, one RCT study found long-term positive results for neurofeedback (*n*=38) compared with computerised attention training (*n*=23), 6 months post-treatment in stimulant-free children with ADHD.^17^ In this study, children for whom stimulant medication was indicated were excluded from the trial or excluded from the follow-up analysis, potentially excluding children with more severe ADHD symptoms. Consequently, the effectiveness of neurofeedback as a treatment for ADHD in this study cannot be generalised to children with more severe ADHD symptoms. A recent published RCT^18^ did overcome this generalisation problem by including children while standard community care continued, including stimulant medication use by 47% of the participants. They found a larger decrease of ADHD problems from pre-intervention to 6-month follow-up for children that received neurofeedback in addition to standard community care (*n*=34) than children receiving only standard community care (*n*=36).^18^ However, significant results were only found on parent reports, which may be biased in this unmasked study. A meta-analysis into the effectiveness of non-pharmacological interventions^15^ indeed showed that direct behavioural effects of neurofeedback reduced to non-significant when only probable masked outcomes were considered. In addition, results of a systematic review^44^ do not support the effectiveness of neurofeedback to enhance neurocognitive functioning.

The effects of comorbid ASD and stimulant medication use and possible interactions with the effects of the treatment over time were also explored in the current study. Adolescents with comorbid ASD did not respond to the interventions differently from adolescents with ADHD only. Analysis of EEG activity at pre-intervention revealed that during resting with eyes open and during task performance adolescents with combined ASD+ADHD showed significant less theta activity than adolescents with only ADHD.^45^ Elevated theta and decreased beta activity in ADHD^10,11,46^ is associated with decreased vigilance and decreased attention^12^ respectively. Accordingly, neurofeedback protocols aim to decrease theta and increase beta and subsequently improve behaviour. Since adolescents with combined ASD+ADHD display less theta activity than adolescent with only ADHD, improvements in behaviour by decreasing theta activity (if possible) using neurofeedback would probably be less pronounced in adolescents with ASD+ADHD than in adolescents with only ADHD. However, in the current study improvements in cognition and behaviour were similar for both diagnostic groups. Similarly, although stimulant-medicated adolescents with ADHD generally display less theta activity than stimulant-free adolescents with ADHD,^34,47–49^ in the current study, use of stimulant medication did not result in different EEG patterns^45^ or intervention outcomes. The absence of specific effects between stimulant-medicated and stimulant-free adolescents might be explained by the fact that the majority of the stimulant-medicated adolescents used stimulant medication for 6 months or longer at study entry,^43^ instead of only acute medication effects that are frequently reported in the literature.

By investigating the long-term effects of neurofeedback as an additional treatment for adolescents with ADHD with a combination of behavioural and neurocognitive measures, we aimed to ensure the ecological validity of the current study. Nevertheless, this also resulted in several limitations. For example, the target population consists of a heterogeneous group of male adolescents with complex problems in a broad age range, and there was variety in the prescription of stimulant medication. To explore possible interactions within this heterogeneous population, we performed *post hoc* analyses for the presence of comorbid ASD and stimulant medication use. It should however be noted that splitting up the treatment groups for these aspects leads to analyses of relatively small subgroups and a subsequent decrease in power for the analyses. Accordingly, conclusions with regard to ASD and stimulant medication should be interpreted cautiously. Further research, comparing neurofeedback with stimulant medication, titrated with a stepwise double-masked placebo-controlled protocol is essential to see whether neurofeedback is able to be a long-term alternative for stimulant medication. Another point of consideration is the absence of masking of the current study. Masking ensures that expectations about the treatment do not create a bias in favour of one of the randomised treatment conditions. However, in the current study, no additional value of neurofeedback was found. Therefore, it seems unlikely that a positive expectation bias has influenced the results. Another limitation is the low response rate for the parent reports at follow-up, which could therefore not be statistically evaluated. Most studies into the effectiveness of neurofeedback included parent reports, sometimes combined with teacher reports on behaviour. Accordingly, the absence of parent reports and use of self-reports decrease the ability to directly compare the current study to former studies. On the other hand, the use of self-reported behavioural data give new information about whether or not participants themselves notice improvements in behaviour over time.

In conclusion, 1 year after treatment the reduction in ADHD symptoms and improvements in neurocognitive performance were similar in adolescents who received neurofeedback in addition to TAU and adolescents receiving TAU alone. These results do not support the use of theta/SMR neurofeedback as complementary treatment for TAU to produce enduring improvements in behaviour or neurocognitive functioning in adolescents with ADHD. Considering the absence of robust additional long-term effects of neurofeedback, combined with the absence of specific effects of neurofeedback over sham neurofeedback^35,44,50^ and the requirement for intensive training (20–40 sessions),^10^ the use of theta/SMR neurofeedback as a treatment for adolescents with ADHD is not supported.
